# The effect of cold water endurance swimming on core temperature in aspiring English Channel swimmers

**DOI:** 10.1186/s13728-016-0044-2

**Published:** 2016-02-01

**Authors:** Tara Diversi, Vanessa Franks-Kardum, Mike Climstein

**Affiliations:** Nutrition and Dietetics, Institute of Health and Sport, Faculty of Health Sciences, Bond University, Gold Coast, QLD Australia; Water Based Research Unit, Bond University, Gold Coast, QLD Australia; Exercise Health and Performance Faculty Research Group, Faculty of Health Sciences, The University of Sydney, Lidcombe, Sydney, NSW Australia

**Keywords:** English Channel, Open water swimming, Cold water swimming, Hypothermia, Dual-energy x-ray absorptiometry, Core temperature, Body fat, Body composition

## Abstract

**Background:**

The purpose of this study was to determine if cold water swimmers (CWS) developed hypothermia over a 6-h cold water endurance swim and whether body composition, stroke rate (SR) or personal characteristics correlated with core temperature (TC) change. Nine experienced male and female CWS who were aspiring English Channel (EC) swimmers volunteered to participate. Subjects aimed to complete their 6-h EC qualifying swim (water 15–15.8 °C/air 15–25 °C) while researchers intermittently monitored TC and SR. Data obtained included anthropometry (height, mass, segmental body composition), training volume and EC completion.

**Results:**

Of the nine swimmers who volunteered, all successfully completed their EC qualifying swim. Six CWS had complete data included in analysis. One CWS demonstrated hypothermia (34.8 °C) at 6-h. TC rate of decline was slower in the first 3 h (−0.06 °C/hr) compared to the last 3 h (−0.36 °C/hr) of the swim. Older age was significantly correlated to TC change (*r* = −0.901, *p* < 0.05) and SR change (*r* = −0.915, *p* < 0.05). Absolute and percentage body fat (BF) were not significantly associated with higher TC. Mean SR over the 6-h swim was 57.8 spm (range 48–73 spm), and a significant (*p* < 0.05) decline in SR was observed over the 6 h (−9.7 %). A strong, positive correlation was found between SR change between 3 and 6 h and TC over the 6 h (*r* = 0.840, *p* < 0.05) and TC from 3–6 h (*r* = 0.827, *p* < 0.05). Seven of the nine participants (77.8 %) in this study successfully completed the EC crossing. Successful EC swimmers swam in the pool and open water (OW); however, they swam significantly [*t* (7) = −2.433, *p* < 0.05] more kilometres (*M* = 19.09 km/wk ± 5.55) in OW than unsuccessful (*M* = 9 km/wk ± 1.41) EC swimmers. There was a significant relationship between EC crossing time and height (*r* = −0.817, *p* < 0.05), but no other variables and EC crossing time.

**Conclusions:**

Cold water endurance swim (CWES) of 6-h duration at 15–16 °C resulted in TC reduction in the majority of swimmers regardless of anthropometry. More research is required to determine why some CWS are able to maintain their TC throughout a CWES. Our results indicate that older swimmers are at greater risk of developing hypothermia, and that SR decline is an indicator of TC decline. Our results also suggest that OW swimming training combined with pool training is important for EC swim success.

## Background

Cold water endurance swims (CWESs) such as the English Channel (EC) crossing, like many endurance sporting events, are increasing in popularity [1–4]. The EC is seen as the pinnacle in endurance swimming [5]. At the shortest distance, the EC spans 33.5 km (20.8 min) [4, 6] from England to France. The average successful cross-channel swimmer takes approximately 13 and a half hours to complete the swim crossing [7]. The fastest recorded time is 6 h and 55 min and was achieved in 2012; the slowest recorded time is 28 h and 44 min, completed in 2010 [7].

Hypothermia is a significant potential risk for open water (OW) swimmers [8–11] and has been recorded at water temperatures as high as 22 °C [12]. Hypothermia is broadly defined by core temperature (TC) of less than 35 °C (<95 °F). To reduce the risk of hypothermia in participants in their events, the Fédération Internationale de Natation (FINA) has established a lower water temperature limit of 16 °C [13] for OW swimming events. Similarly, in events longer than 1500 m, the International Triathlon Union (ITU) has made wetsuits compulsory if the water temperature is below 16 °C [14]. In contrast, for an EC swim to be ratified by the Channel Swimming Association (CSA) or the Channel Swimming and Pilot Federation (CS&PF), regardless of the water temperature [15] swimmers are not permitted to wear a wetsuit and are limited to a standard non-buoyant swimsuit, goggles, ear plugs and one swim cap [15, 16]. The water temperature during the Channel season is usually between 11 and 19 °C, with most swims occurring between 14 and 16 °C [17]. In addition, all swimmers are required to complete a 6-hour qualifying swim in water less than 15.5 °C for CSA-ratified [15] and 16 °C for CS&PF-ratified [17] swims.

The duration of exposure to cold water and the conditions of the EC place swimmers at an increased risk of developing hypothermia [12, 18]. Hypothermia has undesirable physiological responses which have previously been shown to contribute to premature swim termination [11]. As a cold water swimmer (CWS) TC approaches hypothermia, physiological mechanisms (nervous, endocrine, muscular and integumentary systems) are initiated in an attempt to maintain core body temperature.

Minor risks of attempting the EC include feeling cold, nausea, emesis, jellyfish stings, salt mouth, otitis externa and musculoskeletal overuse injuries [19]. There are also major health risks which may be associated with hypothermia including blood flow restriction, pulmonary oedema, increased cardiac afterload, myocardial ischaemia, heart failure and even death [11, 20–22]. Since 1926, eight swimmers have lost their life attempting to swim across the EC, with the two most recent in 2012 and 2013 [23]. Both of these deaths occurred within one mile of completing the crossing.

In 2013, approximately 60 % of EC attempts were successful, highlighting that failure is frequent [24]. In preparation for an EC attempt, aspiring EC swimmers invest significant time training and financial resources to cover pilot boat fees, support crew, coaching and professional advisor fees, and where required to cover flights and accommodation. Many aspirants also increase body weight and/or fat mass to a potentially unhealthy level in preparation for the cold water temperature [15, 19, 25].

Body composition of marathon swimmers has been previously reported as being higher in body fat (BF) and shorter in stature than competitive pool swimmers, and the differences are attributed to OW swimmers usually being of lower standard than pool swimmers [26]. The CSA and CS&PF recommend EC aspirants to increase their body weight and adiposity to increase the safety of their swim in an attempt to reduce the risk of developing hypothermia [4, 6]. In non-elite marathon swims, higher BF may be associated with improvements in body temperature regulation when swimming in cold water [19, 27] and BF acts as an insulator against the cold [9]. It has been reported that athletes with higher subcutaneous fat [9, 22, 28, 29] and higher BMIs [10, 12] are at lower risk of developing hypothermia [22] and are more likely to endure cold water swims for longer periods [22, 30].

Other strategies that have been employed to reduce the likelihood of developing hypothermia include cold water adaptation [31–33] and hypothermic exercise training [20, 34, 35]. Factors that have been previously shown to increase hypothermia risk are older age [36, 37], wind chill [11], motion sickness [38] and local muscular fatigue [11].

Potential disadvantages to gaining weight to an ‘overweight’ or ‘obese’ level of BF may include health risks [39] and detriments to swimming performance [40, 41]. It is well established that being ‘overweight’ is a risk factor for chronic diseases such as type two diabetes mellitus, cancers (i.e. colorectal and breast cancer) and cardiovascular diseases (i.e. atherosclerosis, stroke and coronary heart disease) [39]. However, exercise is known to reduce the risk for these health conditions [39] and some research shows that this is independent of fatness level [42].

To measure swim performance, indicators of swimming velocity, stroke rate and/or stroke length are commonly used in both elite pool swimming [43–45] and cold water swimming [28, 46]. In freestyle swimming, increased SR is associated with increased metabolic heat production and typically results in an increased velocity [47]. Stroke rate is also related to energy output, and thus a decline in SR is generally indicative of a swimmer’s state of fatigue and performance [48].

Given the health, medical, time and financial risks, along with the increasing popularity of the EC swim [4], this study aimed to observe the TC changes in CWS in an EC qualifying swim of 6 h. We investigated whether factors such as adiposity, lean mass, SR, age or training volume affected TC change, and if any of these factors were associated with EC success and EC crossing time.

## Methods

### Subjects

For this study, we aimed to recruit male and female CWS aged 16 years and older who passed an EC medical assessment and would be attempting to swim the EC in the 2014 season. Demographic description of our participants is shown in Table 1. Prior to conducting this study, ethics approval was obtained from the Bond University human research ethics committee (RO1808). All participants provided written informed consent after being advised of the methods and potential risks of involvement in the study. Potential participants with existing gastrointestinal disorders were excluded from participation.

**Table 1:** CWS participant demographics

Parameter	Group (*n* = 9)	Male (*n* = 6)	Female (*n* = 3)
Age (years)	46.0 (±16.8)	45.83 (±15.0)	46.3 (±23.9)
Height (cm)	175.4 (±8.6)	180.13 (±6)*	165.9 (±1.8)
Mass (kg)	82.8 (±9.4)	85.9 (±7.5)	76.4 (±11.2)
BMI (kg/m^2^)	27.0 (±2.4)	26.45 (±1.6)	28 (±3.71)

Mean ± SD; * *p* < 0.01

### Participants’ training demographics

Prior to the swim, the participants were asked to provide their CWS training regimes which included their volume of training identified by total kilometres swam each week and the average kilometres swam in the pool and swam in OW.

### Segmental body composition

Participants’ height and mass were measured using a standard medical balance scale. Segmental body composition was measured via dual-energy x-ray absorptiometry (DXA) (Hologic Discovery A, Waltham, MA). Prior to scanning, the DXA was calibrated with known phantoms to ensure accurate and reliable results. The DXA scanning procedure was performed according to the Australian Institute of Sport protocol [49] which required participants to wear minimal clothing (underwear), fast overnight, void their bladder/bowls prior to scan and avoid any strenuous exercise in the previous 24 h.

### Core temperature

To intermittently monitor body TC, we utilized the Cortemp™ telemetry system (Cortemp, HQInc, Palmetto, Florida) (http://www.hqinc.net/cortemp/products/) consisting of an ingestible sensor which transmits a low-frequency radio wave to a wireless data recorder [50, 51]. Participants were instructed to ingest the pill sensor between 4 and 8 h prior to the 6 a.m. swim start, as this has been shown to allow the sensor to move further into the GI tract providing more accurate readings [51]. Temperature was measured prior to the consumption of food or fluids to reduce the effect of the temperature of the foods affecting the temperature reading [52].

### Six-hour swim

Participants were attempting to complete a scheduled 6-h CWES (water 15–15.8 °C/air 15–25 °C) as an EC qualifying swim. Swimmers could not be touched or assisted by researchers in order for the swim to be accepted as an EC qualifying swim. The swim was completed in a protected part of Sydney Harbour, in calm conditions (no waves, low current, low wind). Prior to entering the water, the pre-swim TC of each participant was recorded. Approximately every hour following commencement of the swim, TC was measured and recorded by an investigator. Swimmer’s TC was recorded while swimmers stopped and floated, refuelling with food and/or liquid. The duration of the stop was not recorded; however, swimmers were encouraged to fuel as quickly as they would in their cross-channel attempt. Given the water temperature exposure, we assume that each swimmer experienced a decrease in temperature due to the lack of metabolic heat production during each stop. However, during a cross-channel attempt, swimmers will be required to make stops to feed, therefore this replicates what would happen during their attempt.

An experienced OW swimming coach who holds a silver licence with the Australian Swimming Coaches and Teaching Association recorded each participant’s SR as this has previously been shown to be an indicator of swimming performance [48]. All participants were under the supervision of their coaches who were allowed to terminate a participant’s swim for any reason. Post-swim TC was recorded at the conclusion of the 6-h swim.

### Hypotheses

The primary aim of this study was to test the hypothesis that TC would decrease over a 6-h CWES in experienced CWS. Additionally, the study tested the hypothesis that TC change would be greater in those with lower BF percentage and increased age. It was also hypothesized that SR decline would correlate with TC decline and those with less adiposity would be more likely to terminate the qualifying swim and EC attempt early than those with higher adiposity. With regard to the EC swim, it was hypothesized that EC crossing time would be correlated to BMI and BF %.

### Analysis

Normality of all data was assessed by investigating kurtosis, skewness and Q–Q plots. Statistical analysis of data included *t* tests (between groups), repeated measures ANOVA (serial time measurements) and Pearson’s bivariate correlations to determine statistical significance. Alpha was set a priori at *p* < 0.05 to determine statistical significance.

## Results

### Participants

A total of nine experienced CWS (six male, three female) aged between 25 and 73 years met all inclusion criteria. Table 2 provides a description of individual participants. All swimmers successfully completed their qualifying 6-h CWES. In three participants, TC readings were recorded until equipment failure (*n* = 2 at 4 and 5 h) and premature excretion of the sensor (*n* = 1 at 4 h) resulted in TC unable to be recorded for the duration of the swim.

**Table 2:** CWES individual participant results

Parameter	CWS Participant								
	1	2	3	4	5	6	7	8	9
Gender	F	M	M	M	M	F	F	M	M
Age (years)	27	70	48	39	25	39	73	51	41
Ht (cm)	168.0	173.0	177.9	183.9	189.0	164.7	165.0	175.0	182.0
Mass (kg)	76.81	74.70	79.36	88.5	95.3	65.1	84.7	89.5	88.4
BMI (kg/m^2^)	26.9	24.9	25.0	26.1	26.7	24.9	32.1	29.2	26.7
Body fat (%)	32.3	17.76	23.4	25.3	19.4	32.9	43.3	22.5	26.5
TC change (over 6 h CWES)	+0.01	−2.31	−1.62	−0.63	+0.042^a^	−1.37	−1.98	+0.03^b^	−0.6^c^
EC swim time (min)	781	765	599	544	558	DNC^d^	DNC^e^	835	684

*DNC* Did not complete

^a^Change over 4 h. Premature excretion

^b^Change over 4 h. Exquipment failure

^c^Change over 5 h. Exquipment failure

^d^Withdrawn after 11 h 20 min

^e^Withdrew after 4 h 3 min

There were no significant differences between genders with regard to age or weight, females were slightly older than males (+1.0 %), males had a higher body mass (+12.4 %) and females had a higher body mass index (BMI +5.8 %).

Self-reported training information was collected from participants. Swimmers in this study reported training an average of 5.38 sessions/week (SD ± 0.49), with weekly total distances ranging from 30 to 55 km (*M* = 42.78, SD ± 7.19). All swimmers trained in the pool and OW, with distances ranging from 18 to 40 km swam in the pool (*M* = 26.67, SD ± 6.63) and 8 to 20 km per week in OW (*M* = 16.84, SD ± 6.57).

### Body core temperature

**Fig. 1:**
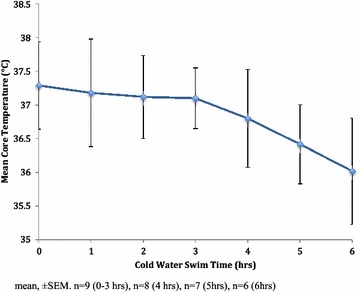
Mean core temperature of swimmers during a 6-h CWES (15 °C–15.8 °C)

The mean pre-swim TC was 37.29 °C (SD ± 0.65) and varied between 36.49 and 38.52 °C (*n* = 9). At the completion of the 6-h swim, TC declined to a mean of 36.02 ± °C (SD ± 0.79) and varied between 34.88 and 37.15 °C ± (*n* = 6) (Fig. 1). Mean post-swim TC was significantly lower (*p* < 0.05) than mean pre-swim TC (mean −0.42 °C, SD ± 1.00) (Table 3).

**Table 3:** Core temperature rate of decline (absolute and percentage)

Time	TC °C (M)	Decrease (°C/hr)	Decrease (%)
Pre CWES	37.29 (n = 9)		
Pre–1 h	37.18 (n = 9)	−0.11	0.30
1–2 h	37.12 (n = 9)	−0.06	0.16
2–3 h	37.10 (n = 9)	−0.02	0.05
3–4 h	36.80 (n = 8)	−0.30	0.81
4–5 h	36.42 (n = 7)	−0.38	1.03
5–6 h	36.02 (n = 6)	−0.40	1.10
Pre–6 h	−1.27	−0.22	3.41
Pre–3 h	−0.19	−0.06	0.51
3–6 h	−1.08	−0.36	2.92

**Fig. 2:**
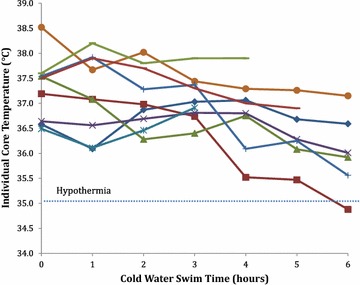
Individual swimmer’s core temperature over a 6-h CWES (15 °C–15.8 °C)

Individual TCs over the 6-h swim are presented in Fig. 2. Over the 6-h CWES, increased age was significantly associated with reduced TC (*r* = −0.901, *p* < 0.05). The two oldest swimmers had the greatest reduction in TC over the swim and the most dramatic drops between 3 h and completion of the swim. To determine whether the reduction in TC was related to performance, correlation between SR and age and EC completion time and age were calculated.

### Body composition

The majority (*n* = 6) of swimmers within this study were overweight according to BMI (BMI 25–29.99 kg/m^2^), one swimmer was classified as obese (BMI 30–34.99 kg/m^2^) and two swimmers were within the healthy weight range (BMI 18.5–24.99 kg/m^2^). The swimmer who developed hypothermia during the qualifying swim had the lowest BMI (24.9 kg/m^2^) and lowest BF percentage (BF %) (17.76 %) of all participants. Males had a significantly lower total BF % (−38.3 %) and lower average leg fat mass (−36.1 %) and arm fat mass (−31.8 %) than females. Although there was a trend towards a correlation between increased BMI and increased BF %, this was not statistically significant (*r* = 0.602, *p* = 0.86) (Table 4).

**Table 4:** CWS participant segmental body composition

Parameter	Group (*n* = 9)	Male (*n* = 6)	Female (*n* = 3)
Total fat mass (kg)	22.74 (±6.2)	20.01 (±2.2)	28.2 (±8.8)
Trunk fat mass (kg)	10.68 (±3.5)	9.5 (±0.8)	13.0 (±6.0)
Arms fat mass (kg)	2.42 (±0.68)	2.2 (±0.4)	2.9 (±1.0)
Legs fat mass (kg)	8.62 (±2.61)	7.25 (±1.6)*	11.34 (±2.1)
Total fat (%)	26.9 (±8)	22.32 (±3.2)	36.2 (±6.2)
Trunk fat (%)	25.93 (±6.8)	22.51 (±1.8)	32.8 (±8.4)
Arms fat (%)	25.92 (±8.5)	20.87 (±2.9)	36.1 (±6.0)
Legs fat (%)	31.62 (±9.6)	25.78 (±4.2)	43.32 (±4.2)
Total lean mass (kg)	58.49(±10.1)	64.16 (±6.1)*	47.2 (±5.1)
Trunk lean mass (kg)	27.9 (±3.6)	31.6 (±1.0)*	25.5 (±1.9)
Arms lean mass (kg)	6.9 (±1.8)	7.97 (±1)**	4.8 (±0.7)
Legs lean mass (kg)	17.9 (±3.6)	19.78 (±2.6)	14.3 (±2.5)

Mean ± SD; * *p* < 0.05; ** *p* < 0.01

The majority of swimmers (*n* = 5) had BF % classified as overweight for their gender and age. Four swimmers had BF % in the acceptable range for their gender and age. There was no significant correlation between BF % (*r* = 0.163, *p* = 0.73) or BMI (*r* = 0.471, *p* = 0.29) and EC swim time. The swimmer who was able to best maintain her TC and SR throughout the swim had a measured BF % of 32.9 %, which is just within the acceptable range for her gender and age. In our study, we found that there were no significant associations between potential measures of performance (SR or EC crossing time) and any BF absolute or % measures.

### Stroke rate

**Fig. 3:**
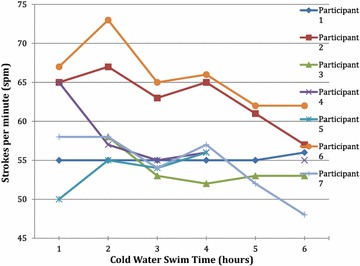
Individual swimmer’s stroke rate over a 6-h CWES (15 °C–15.8 °C)

During the qualifying swim, SR at the first hour ranged between 53 and 67 strokes/min (*M* = 60.5 spm, SD ± 2.5). Individual SRs throughout the swim are presented in Fig. 3. Mean group SR increased slightly (+1.3 %) at the second hour, to a mean of 61.3 strokes/min (SD ± 7.1) before decreasing (−6.2 %, *p* < 0.05) significantly to a mean of 57.5 strokes/min (SD ± 5.1) at 3 h. SR significantly declined between the second and sixth (*p* < 0.05) and second and third hours (*p* < 0.05). No significant correlation was found between the overall change in TC and SR from 1 to 6 h (*r* = 0.471, *p* = 0.346); however, a significant correlation was found between TC and SR change from 3 to 6 h (*r* = 0.827, *p* < 0.05). A significant correlation between age and SR change from 3 to 6 h was also found (*r* = −0.915, *p* < 0.05). No correlations were identified between any overall SR change and total BF % (*r* = −0.386, *p* = 0.522), arm fat mass % (*r* = −0.342, *p* = 0.573), leg fat mass % (r = −0.330, *p* = 0.588), BMI (*r* = −0.221, *p* = 0.674) or height (*r* = −0.081, *p* = 0.879).

### English Channel success

**Fig. 4:**
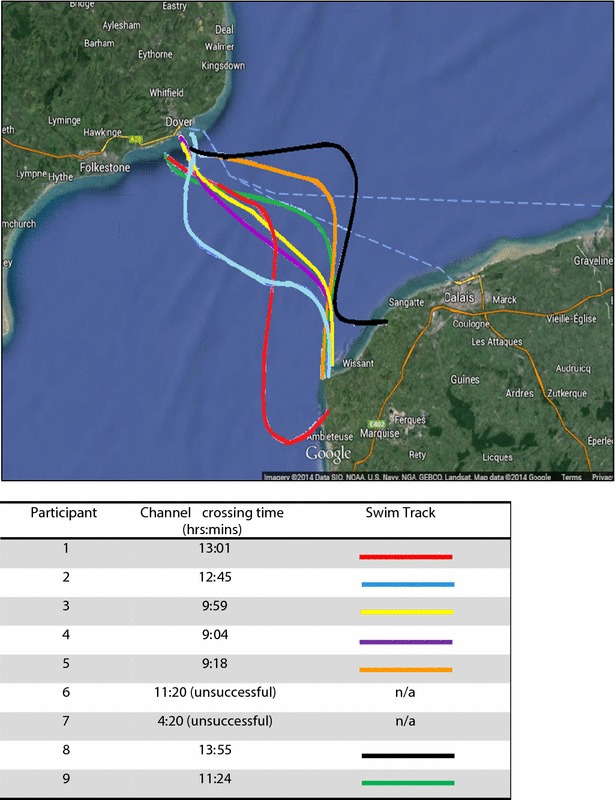
Successful English Channel swimmer’s swim tracks

All participants in this study attempted the EC swim in the 2014 EC season. Seven of nine (77.8 %) participants were successful. Successful swim times ranged from 9 h 04 min to 13 h 55 min (M = 11 h 15 min (SD ± 1.09) which is faster than the 2014 mean EC solo crossing time of 13:53:16 h (SD ± 2:44, *n* = 121) [7]. Figure 4 illustrates the individual swim tracks of the successful EC swimmers highlighting the variations in the routes.

The two participants who were unsuccessful in their EC attempt were both female. Independent samples *t* tests were completed to determine whether any training variables were significantly different between those who were successful or unsuccessful in their EC attempt. There were no significant differences between successful (*M* = 45 ± 5.88) and unsuccessful (*M* = 35 ± 7.07) EC swimmers and total kilometres swam per week [*t* (7) = −2.054, *p* = 0.79]. However, successful (*M* = 19.09 ± 5.55) EC swimmers swam significantly [*t* (7) = −2.433, *p* < 0.05] more of their training in the OW compared with unsuccessful (*M* = 9 ± 1.41) EC swimmers in our study. Besides gender, there were no physiological commonalities between the two participants. One unsuccessful swimmer was 39 years of age and had a BMI of 24.9 kg/m^2^ and a BF of 32.2 %, categorized at the upper end of the ‘healthy’ range for her BMI and within the healthy range for her age and gender BF % according to Gallagher et al. [54]. The other unsuccessful swimmer was 73 years of age and had a BMI of 32.1 kg/m^2^ categorized within the ‘obese’ range and a BF of 43.3 %, categorized within the ‘morbidly obese’ range. Both swimmers had above-average TC change during the qualifying swim of −1.36 and −1.98 °C, respectively. The older swimmer had the second lowest post-swim TC following the qualifying swim (35.56 °C). Although the exact causes of failure are unknown, both swimmers reported harsh conditions (e.g. wind, ‘chop’) interfered with their swim. The younger swimmer was pulled out of the water involuntarily at 11 h and 20 min by her pilot and crew due to conditions and fog. She was suffering from shoulder pain that later required an operation; however, the swimmer was determined to keep swimming. The older swimmer unfortunately suffered asthma-related breathing difficulties and voluntarily withdrew from the swim after 4 h and 2 min.

EC crossing time and successful participants’ heights were significantly correlated (*r* = −0.817, *p* < 0.05). There was no significant correlation recorded between age and EC completion time (*r* = 0.619, *p* > 0.05). There were no significant correlations between the successful EC swim times and other variables collected or calculated within this study.

## Discussion

### Core temperature

This study set out to investigate whether CWS are at risk of hypothermia in a 6-h EC qualifying CWES in water between 15 °C and 16 °C, and to determine the rate of decline of TC. The present study highlighted the significant risk of hypothermia in CWS. The majority of participants in this study saw a decline in TC over the 6-h swim, and it could be inferred that TC would continue to decrease over time. The average EC crossing time was close to 14 h in 2014, and therefore EC swimmers are at risk of developing hypothermia during their swim. There may also be an afterdrop [10] where TC may continue to drop after the completion of the swim, so rewarming and observation is important.

One participant who had complete data maintained TC for the duration of the swim, and two others with incomplete data also had TC that increased at 4 h prior to the premature excretion of the sensor and equipment failure. These three participants did not have attributes that were different from other members of the group or more cold water experience. However, the participant who maintained her TC throughout the swim also maintained her SR throughout the swim.

Our results supported the hypothesis that older CWS would be at higher risk of hypothermia. Older participants were less able to maintain their TC over the 6-h swim. The swimmers with the greatest TC changes, and two lowest TC on completion of the swim were 73 and 70 years of age with 43 and 17 % BF, respectively. Our study included two older athletes who were both aiming to become the oldest person to successfully complete the EC swim. One was successful in his attempt, only for his record to be broken 1 month later. Due to the small sample number, it is unknown exactly why age was related to lower TC in the qualifying swim. There were no physiological commonalities between our older swimmers. One swimmer was low in adiposity, yet the other was high in adiposity. One swimmer was relatively fast (>3.5 km/hr), where the other was relatively slow (<3 km/hr). The successful older swimmer swam the channel over an hour faster than the average recorded crossing time for the season. However, as swimmer’s age increased, the SR change also increased. Older swimmers were less able to hold their SR over a 6-h swim period, and this may have affected their ability to produce metabolic heat.

Although there is limited research on age and hypothermia in CWES, accidental hypothermia is recognised as more common in older individuals (>60 years) [36, 53]. Usually, thermoreceptors will detect TC changes, prompting thermoregulatory responses [20]. Accelerated TC declines in older people are thought to be caused by impaired thermosensitivity to ‘cold stress’, such as cold water immersion [36, 37]. The responsiveness of older individuals to cold exposure was reported in an early study by Krag and Kountz [55]. The researchers exposed 13 ‘old’ (57–91 years) and six ‘young’ (22–36 years) participants to 5–15 °C air temperatures for 120 min, and observed that the older group experienced greater declines in rectal temperatures. Comparably, Falk and colleagues [56] showed eight older adults (55–70 years) who had similar (*p* > 0.05) fitness levels and skinfolds to eight younger adults (21–29 years), experienced significantly (*p* < 0.05) greater declines in rectal temperature, after 5 °C ambient air exposures. Adding to the reliability of this study, the same protocol was performed in a controlled environment (22 °C ambient air temperature), and no statistical differences were found between groups. This implies that older individuals do not ‘normally’ have a lower TC. Older age may impact TC regardless of speed, fitness or adiposity. More research is needed in the field to understand the risk of hypothermia in older swimmers and if there are any factors that can be controlled to reduce these risks.

## 6 hour swim success

Each of the participants successfully completed the scheduled six-hour swim and qualified to attempt to swim the EC. We could therefore not test whether swimmers with different characteristics such as BF % were more likely to prematurely cease the CWES. For two swimmers, this was the longest swim they had completed at the time. Unfortunately, equipment failure or premature excretion of the sensor resulted in incomplete data for a third of our swimmers. This highlights the difficulty of applied research in CWES. Additionally, for some participants in future studies using temperature sensors over a long duration, taking the sensor closer to the commencement of the study may be recommended.

### Body composition

The CSA [6] recommends that EC aspirants have an adequate amount of BF to prevent them from becoming hypothermic. In support, many studies suggest that subcutaneous fat enhances thermal insulation by preventing metabolic heat loss [12, 57, 58]. Subcutaneous fat acts as an insulator to the cold water [58]. It has been suggested [27] that BF is only important for swims in less than 10 °C; however, hypothermia has been recorded in water as warm as 22 °C [12]. Not all successful channel swimmers are overweight or have high BF. One case report of a successful 24-year-old female swimmer in the early 80s reported the body fatness of 20.1 % using ultrasound-derived skinfolds and the Siri equation, with an abdominal skinfold of 13 mm and thigh skinfold of 8 mm [59]. In our study, high BF was not protective against increased TC change. However, the only participant who recorded a TC classified as hypothermia also had the lowest BF %.

Early studies observed that swimmers with more BF were less likely to experience hypothermia [9, 57, 60]. Keatinge [57] observed 12 subjects in 15 °C water and indicated that the rate of TC decline was closely related to the reciprocal of individual skinfold thickness. More recently, Brannigan et al. [12] investigated 109 swimmers, swimming in 19–22 °C water for 5 to 12 h. Of the 75 participants included in data analysis, hypothermia affected 23 % and they concluded that for every whole unit increase in BMI there was a 43 % reduction in hypothermia rates. However, to measure body temperature Brannigan et al. [12] used oral measurement in 24 cases and paired only two with rectal. Oral thermometers are known to underestimate TC by 0.86–1.67 °C [61, 62].

Low subcutaneous fat in cold water endurance swimming has been associated with early swim termination [22, 28]. For example, Keatinge et al. [22] (2001) analysed eight participants, in water ≤11 °C and reported that swimmers with less fat terminated their swims earlier despite not recording lower TC. This introduces the idea that cold tolerance may not always accurately represent TC and factors involved in maintaining TC may be different from those important for cold tolerance. A case study of two CWS [63] with 25.6 and 16.6 % BF and a pool swimmer with 14.4 % BF in 15.0–17.4 °C water observed the swimmer with the greatest BF being able to swim 167 min longer than the thinner CWS and 222 min longer than the pool swimmer.

Our study found no significant association between body composition and TC. Castro, Mendes and Nobrega [31] found no association between the BF percentage and TC of 12 swimmers over a 10-km swim in 19 °C water. They recorded a much higher incidence of hypothermia in their swimmers (58.3 %), most likely related to the experience of their swimmers.

Other studies suggest arm fat, in particular, may be more indicative of hypothermia risk (and subsequent swim failure) [29, 35, 46]; this is a result of peripheral muscular cooling. Lounsbury and Ducharme (2008) [46] investigated seven inexperienced and eight experienced swimmers swimming in 10 °C water, and observed a positive correlation between the length of time the swimmer could continue swimming and tricep skinfold thickness, regardless of experience. Similarly, Knechtle and colleagues [29] indicated that a swimmer with a greater BF percentage and tricep skinfold thickness, despite having a lower BMI, was able to swim in 4.3 °C water, for longer than another participant (42 vs 23 min). However, as with many studies in the area, results were limited by sample number and potential subject bias, as the participant with greater tricep skinfolds had attempted an Antarctic swim previously. This also introduces the concept that swimmers with more BF and cold water swimming experience may be better equipped psychologically to successfully cross the EC. Hence, the replication of this study with a larger sample group would be beneficial to support or reject recommendations that BF, tricep skinfold thickness and cold water experience may act as hypothermia deterrents. In a study [29] of two swimmers with differing experience and BMIs (27 kg/m^2^ and 23.4 kg/m^2^) but similar BF percentage (21 and 23.4 %) in an ice cold swim (water temp 4 °C), the more experienced swimmer with slightly higher BF percentage was able to swim twice as long as the swimmer who was inexperienced and had a higher BMI, yet lower BF percentage. Overall, BF skinfolds were only an average of 1.9 mm difference per site; however, the chest skinfold (5.6 v 15.4 mm), the front thigh (11 v 15 mm) and the tricep (7.4 v 10.0 mm) skinfold had higher-than-average differences [29] indicating that site-specific skinfolds may be important when trying to identify potential body composition differences in cold water OW swimmers.

Although research comparing body composition and performance in CWES is limited, Knechtle et al. [27] analysed 63 subjects at 21–27 °C and found that swimming speed, BMI and BF % were not associated. Our study also found no correlation between EC time and anthropometric variables except for height.

Increased BMI was not correlated with TC change in our study. BMI may not be protective against TC changes in CWS; however, research with a larger sample and participants with a greater range of body compositions is warranted. Swimmer’s BMI in this study was consistent with previous research where the majority of CWS were overweight (BMI 25–29.99 kg/m^2^) [1, 12, 64] as classified by the WHO. Nuckton and Kohn (2012) [1] measured the BMI of 88 CWS and reported a mean BMI of 25.8 kg/m^2^. These results are similar to Weitkunat et al.’s findings [64] that the mean BMI of 36 CWS was 25.1 kg/m^2^. The ‘overweight’ status of the CWS, derived from BMI, and specific BF percentages from DXAs were not significantly correlated, and because the study was with athletes [65, 66], the BMI should be used with caution despite its convenience over more accurate measures such as surface anthropometry or segmental body composition analysis as used in this study. In our participants, BMI was not significantly correlated to performance as identified by SR changes and EC crossing time. It would be premature to infer that in amateur yet experienced CWS an increased BMI does not negatively affect performance because the majority of the participants in this study had BMIs within 2 kg/m^2^ of each other.

Related to thermoregulation, there are three areas that warrant further investigation. Although it is recommended that swimmers aiming to cross the EC increase body weight and fat levels, there is currently limited evidence base to allow athletes to make informed decisions on how much weight they should gain, or whether it is required based on their personal or swimming characteristics. Secondly, the role of weight gain and its effect on factors other than thermoregulation (i.e. psychological factors such as confidence or cold tolerance). Excessive body weight may have a negative effect on health, yet physical activity increases health. Therefore, for this group which deliberately increase their weight while increasing their physical activity, it would be interesting to further investigate overall health of aspiring English Channel swimmers.

### Stoke rate

For four swimmers, there was an increase in SR between the beginning of the swim and the first hour. This may be in response to the stimulus of cold water and in an effort to increase metabolic heat. It may also simply be related to the swimmers “warming up” into a comfortable rhythm to complete the swim. Tipton et al. [67] measured swimming efficiency and TC in breaststrokers with no previous cold water swimming experience in 10, 18 and 25 °C water. In this study, all 10 participants were able to complete a 90-minute swim at 25 °C; one participant was pulled out of the water with hypothermia in the 18 °C trial, and four participants were withdrawn due to rectal temperatures at or below 35 °C in the 10 °C trial. Swim efficiency was able to be maintained by the swimmers in the 18 °C trial, but not in the 10 °C trial. Just prior to swim failure, swimmer’s SR increased and the stroke length reduced demonstrating poorer swim efficiency and merely continuing with the aim of keeping their head above water [67].

### English Channel success

Seven of the nine swimmers in this study were successful in their feat of becoming EC swimmers. Those swimmers who were successful completed an average of 45 km of swimming training per week. Of this, an average of 19 km was completed in OW. Those who were unsuccessful completed less training (35 km) per week, although this was not statistically significant; however, they completed significantly less training in OW (average of 9 km/week). This highlights the potential importance of training in OW for success in EC crossings.

The swim track of successful swimmers varies depending on the time the athlete starts their swim, the speed of the swimmer, the conditions (tidal movement, wind speed, direction and wave movement) and the skill of the pilot. If a swimmer starts at high tide, they will be pushed in a northeasterly (NE) direction for approximately 4 h on the “flood tide”. Thereafter, there will be a short period of “slack water” as the tide turns. The swimmer will then be pushed in a southwesterly direction for the next 6.5 h, approximately, on the “ebb tide”. Again, there will then be a short period of slack water. Typically, the effects of the tidal movement in this scenario will largely cancel out, but if a swimmer has not landed at Cap Gris Nez (CGN) by the time the next flood tide commences, then they will be pushed in an NE direction again. The difficulty here is that the land falls away on either side of CGN, so even though the swimmer may be swimming in a southeast direction their body will be moving in an NE direction and so will be no closer to France. Due to the movement of the water, swimmers are assisted when travelling further distances, and swim times can be compared as a marker of performance.

The only variable in this study to correlate with EC completion time was height. Taller OW swimmers swam faster across the EC. In a previous study specific to marathon and ultra-marathon swimmers, height was not correlated with performance measures [27]. However, height has previously been associated with pool swimming performance in developing athletes [68], in breaststrokers [69] and in masters swimmers [70]. It is unfortunate that height is one body composition variable that cannot be manipulated through dietary or exercise interventions in adulthood.

## Limitations

Applied research in CWS poses a number of difficulties. Limitations of this study include the small sample size. Completing a 6-h swim in water between 15 °C and 16 °C is a high burden on participants, and can be a high risk if swimmers are not healthy and experienced. For this reason, the convenience sample of swimmers already completing the CWES was chosen. In OW, the conditions and weather change throughout a swim. This represents the unexpected conditions of EC swimming. Because this study was an observational design, and the stakes for the swim being high for some swimmers involved, the participants did not have standardised diet, nutrition or hydration throughout the swim and these may have affected TC changes in addition to those factors we investigated.

## Conclusions

The results of this small study indicate that there is a TC reduction in the majority of CWS in a CWES of 6-h duration at 15–16 °C. These reductions in TC are irrespective of individual’s body composition. More research is required to determine why some CWS are able to maintain their TC throughout a CWES. Our results indicate that older swimmers are at greater risk of developing hypothermia, and that SR decline is an indicator of TC decline. Our results also suggest that OW swimming training is important for EC swim success.

## Abbreviations

BF: body fat
BMI: body mass index
CSA: Channel Swimming Association
CS&PF: Channel Swimming and Piloting Federation
CWES: cold water endurance swim
CWS: cold water swimmer
DXA: dual-energy x-ray absorptiometry
EC: English Channel
SR: stroke rate
TC: core temperature
WHO: World Health Organisation
