# Erratum to: The effect of cold water endurance swimming on core temperature in aspiring English Channel swimmers

**DOI:** 10.1186/s13728-016-0045-1

**Published:** 2016-02-24

**Authors:** Tara Diversi, Vanessa Franks-Kardum, Mike Climstein

**Affiliations:** Nutrition and Dietetics, Institute of Health and Sport, Faculty of Health Sciences, Bond University, Gold Coast, QLD Australia; Water Based Research Unit, Bond University, Gold Coast, QLD Australia; Exercise Health and Performance Faculty Research Group, Faculty of Health Sciences, The University of Sydney, Lidcombe, Sydney, NSW Australia

## Erratum to: Extrem Physiol Med (2016) 5:3 DOI 10.1186/s13728-016-0044-2

It has come the publisher’s attention that the original version of this article [1] unfortunately contained an incorrect value in the Results section of the text. In the last line of the paragraph Body Composition, the *p* value should read 0.086 and not 0.86.
